# B-Type Natriuretic Peptide at Admission Is a Predictor of All-Cause Mortality at One Year after the First Acute Episode of New-Onset Heart Failure with Preserved Ejection Fraction

**DOI:** 10.3390/jpm12060890

**Published:** 2022-05-28

**Authors:** Liviu-Nicolae Ghilencea, Gabriel-Cristian Bejan, Marilena-Brîndusa Zamfirescu, Ana Maria Alexandra Stănescu, Lavinia-Lucia Matei, Laura-Maria Manea, Ismail Dogu Kilic, Serban-Mihai Bălănescu, Andreea-Catarina Popescu, Saul Gareth Myerson

**Affiliations:** 1 Department of Cardiology, Elias University Hospital, Carol Davila University of Medicine and Pharmacy, 011416 Bucharest, Romania; brindusa.zamfirescu@yahoo.com (M.-B.Z.); amarielavinia@yahoo.com (L.-L.M.); manea.laura.maria@gmail.com (L.-M.M.); smbala99@hotmail.com (S.-M.B.); andreea.catarina.popescu@gmail.com (A.-C.P.); 2 Department of Family Medicine, Carol Davila University of Medicine and Pharmacy, 011227 Bucharest, Romania; cristian.bejan@umfcd.ro; 3 Department of Cardiology, Pamukkale University Hospital, Denizli 20160, Turkey; idogukilic@gmail.com; 4 Division of Cardiovascular Medicine, Radcliffe Department of Medicine, University of Oxford, Oxford OX3 9DU, UK; saul.myerson@cardiov.ox.ac.uk

**Keywords:** NT-proBNP at admission, acute heart failure, preserved ejection fraction, all-cause mortality, risk stratification, estimated glomerular filtration rate, left atrial volume index, TAPSE, atrial fibrillation

## Abstract

Background: Heart failure with preserved ejection fraction (HFpEF) has been assessed extensively, but few studies analysed the predictive value of the NT-proBNP in patients with de novo and acute HFpEF. We sought to identify NT-proBNP at admission as a predictor for all-cause mortality and rehospitalisation at 12 months in patients with new-onset HFpEF. Methods: We analysed 91 patients (73 ± 11 years, 68% females) admitted for de novo and acute HFpEF, using the Cox proportional hazard risk model. Results: An admission NT-proBNP level above the threshold of 2910 pg/mL identified increased all-cause mortality at 12 months (AUC = 0.72, sensitivity = 92%, specificity = 53%, *p* < 0.001). All-cause mortality adjusted for age, gender, medical history, and medication in the augmented NT-proBNP group was 16-fold higher (*p* = 0.018), but with no difference in rehospitalisation rates (*p* = 0.391). The predictors of increased NT-proBNP ≥ 2910 pg/mL were: age (*p* = 0.016), estimated glomerular filtration rate (*p* = 0.006), left atrial volume index (*p* = 0.001), history of atrial fibrillation (*p* = 0.006), and TAPSE (*p* = 0.009). Conclusions: NT-proBNP above 2910 pg/mL at admission for de novo and acute HFpEF predicted a 16-fold increased mortality at 12 months, whereas values less than 2910 pg/mL forecast a high likelihood of survival (99.3%) in the next 12 months, and should be considered as a useful prognostic tool, in addition to its utility in diagnosing heart failure.

## 1. Introduction

Heart failure with preserved ejection fraction (HFpEF) is a phenotype of heart failure (HF), defined by a left ventricular ejection fraction (LVEF) ≥50%, in the presence of symptoms and signs of heart failure and structural and/or functional cardiac abnormalities or raised natriuretic peptides [1]. HFpEF remains a diagnostic challenge due to considerable heterogeneity in clinical presentation and difficulty determining a cardiac cause for the symptoms. HFpEF can present acutely, requiring immediate medical attention [1,2]. There are also therapeutic challenges, owing to the paucity of treatment options with convincing evidence for morbidity or mortality reduction [1,3,4]. Despite the advances in treatment, acute heart failure (AHF) is still associated with increased morbidity and mortality, high rates of rehospitalisations, and a significant financial burden [2,4].

Whether it is described with the name of de novo and acute [5,6], or as new-onset acute heart failure (NOAHF) [7,8], this phenotype represents the same entity of patients with a first acute episode of decompensation of newly-discovered heart failure; in other words, a heart failure that begins with an acute episode. New-onset (de novo) acute heart failure (NOAHF) and chronic decompensated heart failure (CDHF) are two different groups, as the patients with NOAHF show a different clinical profile from patients with CDHF. There is contradictory evidence regarding the prognosis between the first acute episodes of new-onset (de novo) heart failure and the decompensation of chronic heart failure [5,7,8].

The first episode of acute HFpEF with new-onset could be considered a key moment in the course of the disease. Often, it represents the first opportunity to make a diagnosis, and initiate treatment. Certain patient characteristics could indicate a worse prognosis with frequent future hospitalisations. Few studies have examined prognostic data in patients with de novo and acute heart failure, but there is a renewed interest in this issue [5,6,7,8]. Many different biomarkers represent potential predictors, including the plasma concentration of N-terminal-pro-B-type Natriuretic Peptide (NT-proBNP). They are part of the oxidative stress (uric acid, myeloperoxidase), myocardial injury (troponin, creatinine kinase, H-type fatty acid binding protein), renal injury (creatinine, cystatin C, electrolytes, neutrophil gelatinase associated lipocalin), fibrosis matrix remodeling (suppression of tumourigenicity-2, galectin 2, matrix metalloproteinase), inflammation (high sensitivity C-reactive protein, interleukin-6, suppression of tumourigenicity-2, tumour necrosis factor-alpha, procalcitonin), chamber dilatation myocardial stretch (natriuretic peptides), and neuroendocrine processes (copeptin, endothelin-1) [9]. NT-proBNP has a well-established diagnostic and prognosis value in patients with acute heart failure due to reduced ejection fraction, and is still widely studied in different clinical settings as a risk predictor, whereas old and new insights in heart hemodynamics prove that clinical signs of congestion have a delay of up to three weeks after increasing the pressure in the left atrium [10,11,12,13,14,15,16].

The current study aimed to assess the ability of NT-proBNP at admission to predict mortality risk at one year in patients with a first acute episode of new-onset HFpEF, and to subsequently identify the predictors that may explain the augmented NT-proBNP above the calculated and internally validated threshold.

## 2. Materials and Methods

### 2.1. Study Design

For this prospective observational study, we assessed 125 consecutive patients hospitalised at Elias University Hospital (EUH) with an initial first episode of acute HFpEF (based on their primary electronic patient record, EPR) between April 2017–October 2019. Demographic, clinical, biochemical, and echocardiographic data were collected and used for further analysis.

We recruited consecutive patients with: acute dyspnea of cardiac origin as the main reason for hospital presentation (with clinical signs and symptoms of HF, according to the Framingham criteria) [17], requiring first-time hospitalisation, LVEF ≥ 50% [1] (assessed by echocardiography with the modified Simpson’s rule), N-terminal pro-B type natriuretic peptide (NT-proBNP) at admission >300 pg/mL (in sinus rhythm) and >600 pg/mL (in atrial fibrillation, AF) [18,19], and at least one additional criterion: diastolic dysfunction (defined as at least three of the following: average E/e’ratio > 14, septal e’ velocity < 7 cm/s or lateral e’ velocity < 10 cm/s, tricuspid regurgitation velocity > 2.8 m/s, left atrial volume index (LAVI) > 34 mL/m^2^ or left ventricle mass index (LVMI) ≥ 115 g/m^2^ for males and ≥95 g/m^2^, for females).

Patients with other conditions that could cause shortness of breath and interfere with the production of NT-proBNP (acute coronary syndromes, acute pulmonary embolism, pericardial constriction and obstructive hypertrophic cardiomyopathy, significant left heart valve disease [moderate or severe stenosis, severe regurgitation]), or interfere with the washout of NT-proBNP (stage 4 or 5 chronic kidney disease) were excluded. Patients lost to follow-up due to poor compliance or to the COVID-19 pandemic outbreak were also excluded from the analysis.

We have chosen to test the prediction ability of NT-proBNP at admission, for mortality and rehospitalisation, bearing in mind the practicability and convenience of such a predictor, as it is already a widely-used blood test in the diagnosis of HF. Further on, we identified a threshold for NT-proBNP, with values on the high side that would indicate patients with acute HFpEF and higher mortality at 12 months. As a third step of the research, the demographic, biological, and echocardiographic parameters were tested for predictors of high-set NT-proBNP.

All patients included in the study have given their written informed consent. The ethics committee of EUH has given its approval for the investigation, in line with the principles outlined in the Declaration of Helsinki [20].

### 2.2. Statistical Analysis

Data for continuous variables are presented as mean ± standard deviation (SD) or as median and interquartile range (IQR) depending upon the Gaussian distribution. Categorical data are reported as numbers and percentages (%).

We performed comparisons of the central tendency (mean or median) of the continuous characteristics with Student’s *t*-test and with nonparametric tests (Mann–Whitney U rank-sum test), according to their distribution. Categorical data are compared using Pearson’s chi-squared test. All *p*-values were two-sided, and a *p*-value < 0.05 was considered statistically significant.

We used the Cox proportional hazard risk model to test NT-proBNP as an independent predictor for mortality (area under the ROC curve ≥0.60 and a Hosmer–Lemeshow value ≥0.05); the optimum cut-off value was calculated with the receiver operating characteristic (ROC) analysis using the Youden J statistic to maximise the sensitivity and specificity [21]. The study cohort was split into two groups, according to NT-proBNP values at admission: low-level (beneath the cut-off) and high-level (above the cut-off) groups. The Kaplan–Meier method was performed to create survival estimates of the prognosis of patients with low- and high-risk scores, and a Log-rank test was assessed to prove good discrimination of survival times between the two groups. Patients were censored at the time of death, outcome of interest, or last available follow-up, whichever came first. The mortality risk was adjusted for age, gender, comorbidities, and medication with multivariable proportional hazard regression.

The significantly different variables between the high- and low-set groups were assessed for predictors with binary logistic regression. The goodness-of-fit tests were run (AIC, BIC) to choose the best model. Cut-off characteristics were reported using sensitivity, specificity, positive predictive value (PPV), negative predictive value (NPV), accuracy, positive likelihood ratio (+LR), and negative likelihood ratio (−LR).

The results were internally validated by using a randomised contingent (*n* = 45 patients) from the initial training cohort (24 patients with NT-proBNP over the threshold, and 21 patients below the threshold), with the same ratio as the training cohort (1.16:1). Both groups were compared with C-index by analysing the AUROC curves, and the inter-ROC curve difference with the Hanley–McNeil test.

Although the number of patients included in this study is relatively small, we consider the results relevant based on the thorough statistical methods applied. We have used G*Power software, version 3.1.9.7 (Heinrich-Heine-Universität, D-40225 Düsseldorf, Germany) to assess the power of the statistical method applied to our dataset, with α = 0.05 and power (1–β) = 0.95 [22,23].

The statistical analysis was performed with the SPSS (Statistical Package for the Social Sciences) software, version 26 (IBM SPSS Statistics, IBM Corp., Armonk, NY, USA).

## 3. Results

### 3.1. Patient Population

**Figure 1:**
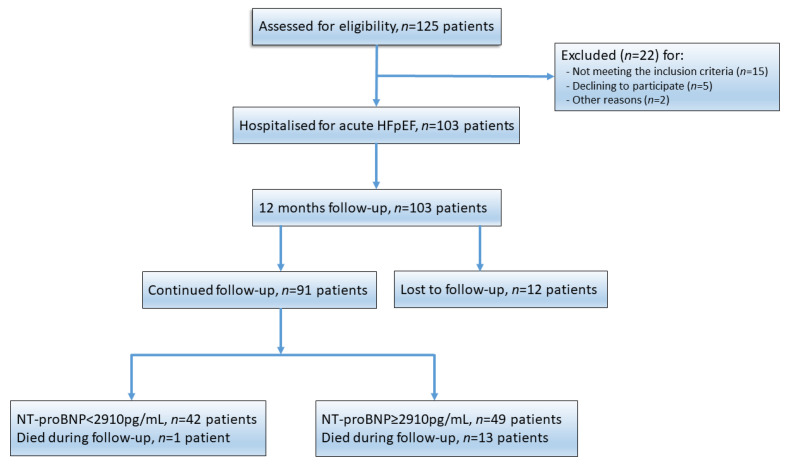
Flowchart of the study.

The analysed population consisted of 91 patients (73 ± 11 years, 68% females) hospitalised in EUH for the first episode of acute HFpEF. Within 12 months of the first acute hospitalisation, 14 patients died, and 49 patients required rehospitalisation (Figure 1).

### 3.2. Baseline Characteristics

The cohort was characterised by an increased frequency of arterial hypertension (100%), atrial fibrillation (69.2%), obesity (59.3%), diabetes mellitus (DM, 56%), and chronic kidney disease (CKD, 43.9%). Other additional conditions were diagnosed: coronary artery disease (CAD, 23.1%, with more than 50% stenosis, in more than one vessel), cerebrovascular disease (19.8%), sleep apnoea syndrome (13.2%), chronic obstructive pulmonary disease (COPD, 13%), and asthma (8.7%).

At the admission, 90% of patients needed supplemental oxygenation, 22% of them needed non-invasive ventilation, and 6% needed mechanical ventilation (Table 1).

### 3.3. NT-proBNP—Independent Predictor for Mortality at 12 Months

The median baseline NT-proBNP value for the entire cohort was 3074 pg/mL (IQR = 5241). The baseline NT-proBNP at admission correlated with 12-month all-cause mortality (r = 0.27, *p* = 0.009), but not with all-cause rehospitalisation (r = 0.18, *p* = 0.104).

**Figure 2:**
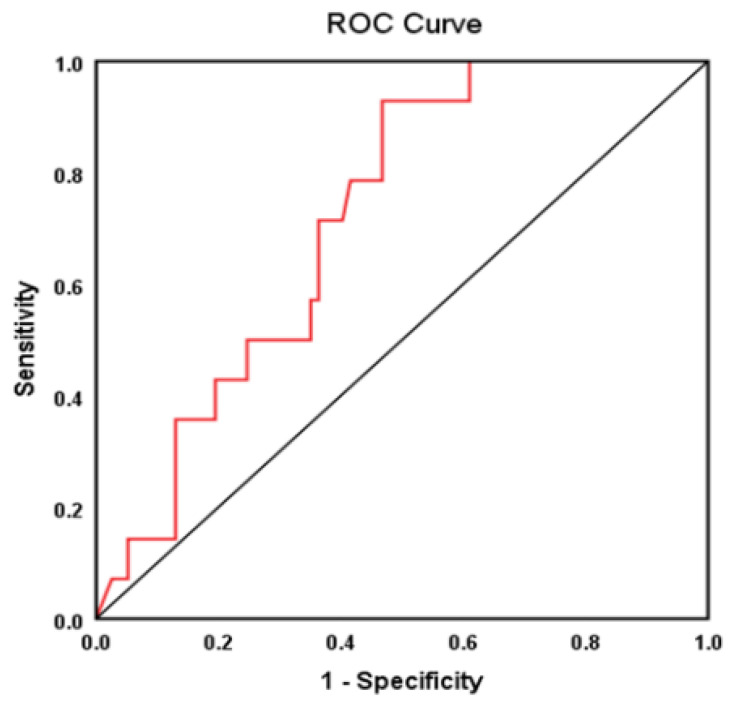
NT-proBNP as a predictor of all-cause mortality at 12 months from the first episode of HFpEF. ROC = Receiver Operating Characteristic.

According to the Cox proportional hazard risk model, NT-proBNP is an independent predictor of all-cause mortality 12 months after a de novo and acute HFpEF event (Hosmer–Lemeshow test *p* = 0.148, AUROC = 0.72, 95% CI = 0.60–0.84, *p* = 0.009) (Figure 2).

The Cox regression equation for our model (NT-proBNP) is:

Log_e_ Odds ratio (Death of all-causes at 12 months) = 2.558 + 12.907 × (NT-proBNP)

An internal validation of NT-proBNP as a predictor of all-cause mortality at 12 months was performed by comparing the training cohort (91 patients, AUROC = 0.72, 95% CI = 0.60–0.84, *p* = 0.009) and a validation contingent (45 patients, AUROC = 0.74, 95% CI = 0.57–0.90, *p* = 0.01). The curves show good superposition and no significant differences (Hanley and McNeil test, *p* = 0.82) (Figure S1).

### 3.4. NT-proBNP Threshold for 12-Month Mortality

**Figure 3:**
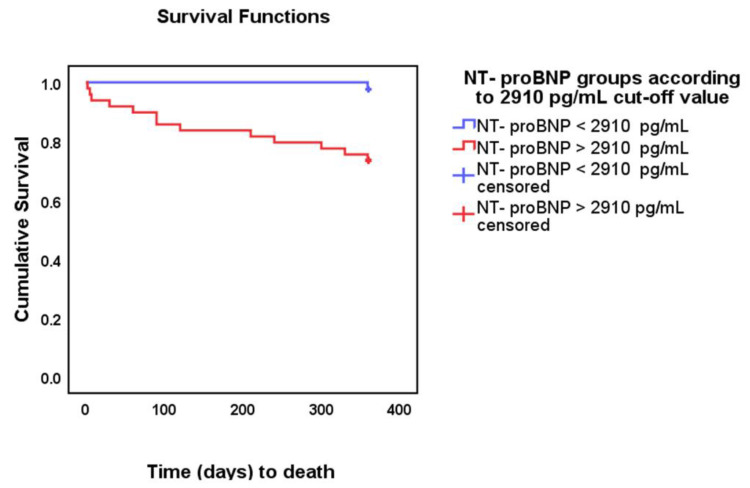
Survival curves for all-cause mortality at 12 months’ follow-up in patients with de novo acute hospitalised HFpEF. Kaplan–Meier cumulative survival in the two groups: NT-proBNP < 2910 pg/mL (blue), and NT-proBNP ≥ 2910 pg/mL (red) (time in days to death).

The optimal NT-proBNP threshold for predicting all-cause mortality in the first 12 months was 2910 pg/mL (sensitivity = 92%, specificity = 53%) (Figure 3). Patients with NT-proBNP < 2910 pg/mL had a 99.3% likelihood (negative predictive value) of survival in the next 12 months (95% CI = 99.5–99.9%). This threshold was used to examine the differences in mortality between the low NT-proBNP group (<2910 pg/mL, *n* = 42) and the high (augmented) NT-proBNP group (≥2910 pg/mL, *n* = 49). Patients with high NT-proBNP had an unadjusted mortality risk that was 12.9 times higher than the patients with low NT-proBNP for all-cause mortality at 12 months (95% CI = 1.7–98.7, *p* = 0.014). The risk of death at 12 months, adjusted for age and gender, was 10.3 (HR = 10.3, 95% CI = 1.34–80.2, *p* = 0.025), whereas the hazard ratio adjusted for medication was 16.1 (95% CI = 1.7–148.5, *p* = 0.014). The risk of death at 12 months, adjusted for age, gender, history of AF, diabetes mellitus, stroke, myocardial infarction, CKD, lung disease, and for medication, was 16.6 (HR = 16.6, 95% CI = 1.6–169, *p* = 0.018). The Kaplan–Meier survival curves illustrated the difference, and the log-rank test proved good discrimination of survival times between the two groups (*p* = 0.014) (Figure 3).

### 3.5. All-Cause Rehospitalisation and Mortality

The rehospitalisation of patients due to cardiovascular causes at 12 months was similar across the groups (42.9% vs. 53.1%, *p* = 0.33). However, the median time (days) until readmission was shorter in the high-level NT-proBNP group (30 vs. 75, *p* = 0.001).

All-cause mortality at 12 months was higher in the high NT-proBNP group compared with the low NT-proBNP group (26.5%, vs. 2.4% respectively, *p* = 0.001), and with a shorter time (days) to death (90 vs. 360 respectively, *p* = 0.002).

### 3.6. Clinical and Echocardiographic Outcomes

The details of all demographic and echocardiographic data for both NT-proBNP groups are presented in Table 2 (and Table S1).

Several demographic characteristics were found to differ between the two groups—the high-level NT-proBNP group were slightly older (75.3 vs. 70.4 years; *p* = 0.026), had a lower proportion of hypercholesterolemia (71.4% vs 95.2%; *p* = 0.003), and a higher proportion of atrial fibrillation (81.6% vs. 54.8%; *p* = 0.002). Patients with augmented NT-proBNP also had reduced renal function: eGFR (58.0 vs. 76.4, *p* = 0.002).

The groups were comparable for clinical presentation, the presence of coronary artery disease, and almost all echocardiographic parameters—the high-level NT-proBNP group had marginally lower right ventricular TAPSE (19.0 mm vs. 20.9 mm; *p* = 0.017), and larger left and right atrial volumes.

### 3.7. Predictors of an High-Set NT-proBNP (≥2910 pg/mL)

**Figure 4:**
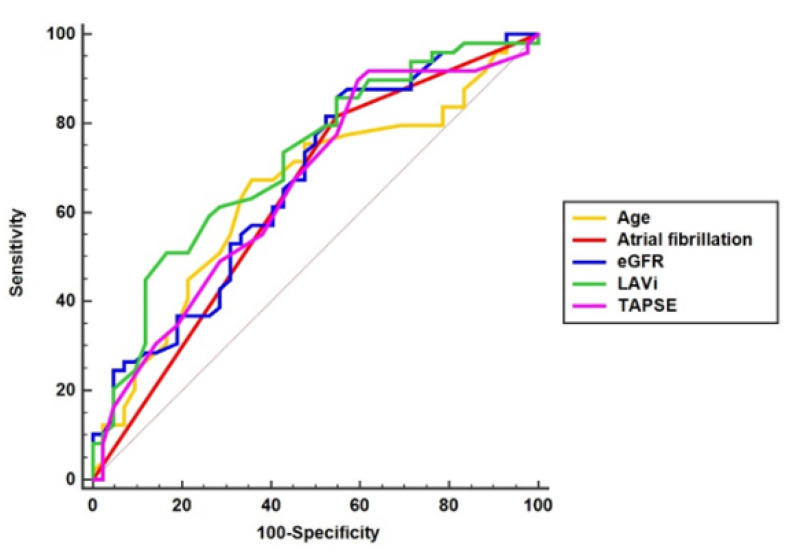
Predictors for raised NT-proBNP ≥ 2910 pg/mL.

The five predictors for raised NT-proBNP in the cohort were: age (AUROC = 0.65, *p* = 0.010), atrial fibrillation (AUROC = 0.64, *p* = 0.028), eGFR (AUROC = 0.67, *p* = 0.006), TAPSE (AUROC = 0.66, *p* = 0.009), and LAVi (AUROC = 0.71, *p* = 0.001), as length of in-hospital stay (AUROC = 0.61, *p* = 0.08), hypercholesterolemia (AUROC = 0.61, *p* = 0.05), IVC collapse < 50% (AUROC = 0.61, *p* = 0.06), right atrium area > 18 cm^2^ (AUROC = 0.61, *p* = 0.06), and stroke volume did not meet the criteria (Figure 4, Table S2).

**Figure 5:**
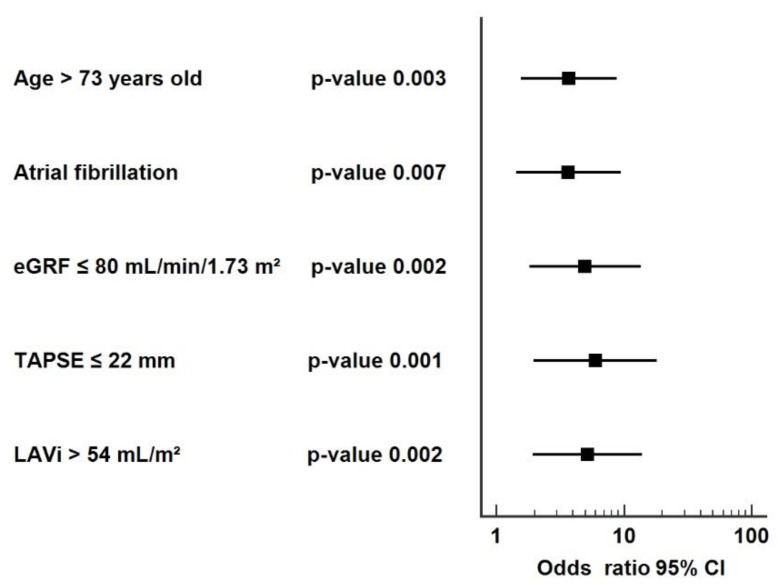
Forest plot for the predictors of NT-proBNP ≥ 2910 pg/mL. The point estimate represents the odds ratio for each predictor; whiskers indicate a 95% confidence interval (CI).

The risks (OR) for an increased NT-proBNP ≥ 2910 pg/mL are: 3.7-fold (*p* = 0.003) for patients aged >73 years, 3.7-fold (*p* = 0.007) for a medical history of atrial fibrillation, 4.9-fold (*p* = 0.002) for eGFR ≤80 mL/min/1.73 m^2^, 5.9-fold (*p* = 0.001) for TAPSE ≤ 22 mm, and 5.2-fold (*p* = 0.002) for LAVi ≥ 54 mL/m^2^, compared with values below the predictors’ thresholds (Figure 5, Table S3).

Although the study cohort is relatively small (91 patients), a post hoc analysis of two-tailed logistic regression, for a total sample of 91 patients, with HR = 10 and α = 0.05, gives a statistical power at 12-month assessment (1–β) of 99.95%.

## 4. Discussion

There is an increasing interest in HFpEF in the medical literature, as risk stratification in acute settings could represent an important tool in therapeutic decision-making [4,5,6,7,8,9,12,13,14,15,16,24,25,26,27,28,29,30,31,32,33,34,35,36,37,38,39,40,41]. However, most studies that have proposed risk scores or predictors for mortality in acute HF investigated mostly patients with heart failure due to reduced ejection fraction.

Our study is original, due to the selection of the patients; to the consideration of the NT-proBNP at admission, rather than at discharge, as a predictor of the 12-month mortality; and to the calculated threshold for high risk.

The cohort selection was very strict and included only patients in acute settings, and with a first recorded episode of heart failure (acute and de novo), which made the group very homogenous as it did not rely on the existing scores in the literature. The choice of this strict selection of patients came from our belief based on observations that the first episode (de novo) of HFpEF represents a turning point in the course of the disease, from which one can deduce valuable information regarding the prognosis.

The results support the idea that NT-proBNP ≥ 2910 pg/mL at admission is an independent predictor, with good sensitivity and specificity, for mortality at one year, in newly-discovered patients with HFpEF. We also identified predictors that increase the NT-proBNP above the validated threshold for these patients with acute and de novo HFpEF.

**Interpretation of the results, and comparison with other studies.**

Our practical approach to an acute episode of de novo HFpEF is based on the assessment of the NT-proBNP at admission, rather than at discharge. Although in our study, a high-set NT-proBNP did not forecast rehospitalisation at one year, it predicted all-cause mortality at 12 months and the time to readmission. Although there is a very good sensitivity, as a value of NT-proBNP ≥ 2910 pg/mL identified 92% of those who died within 12 months, the specificity is low—a NT-proBNP < 2910 pg/mL identified only 53% of the patients who survived 12 months. As a consequence of this low specificity, an NT-proBNP < 2910 pg/mL cannot rule out all-cause mortality at 12 months. An NT-proBNP at the admission of ≥2910 pg/mL predicted an adjusted 16-fold increased risk of mortality in the next 12 months, which is substantially higher than the risk values cited in the literature associated with this test. An NT-proBNP level < 400 pg/mL is commonly used to indicate that a diagnosis of heart failure is less likely, whereas an NT-proBNP level > 400 pg/mL increases the suspicion of heart failure [24]. The prognostic value of BNP has not been well studied, however.

A reference article by Gheorghiade et al. from 2010 proposed a method to quantify the amount of congestion present, by re-assessing the NT-proBNP at discharge as a measurement of grading congestion, to avoid early readmission [12]; although, it admitted that the utility of NP levels may be limited by the fact that their production and release may lag behind acute changes in hemodynamic measurements [13]. Two recent papers published independently by Di Mario [14] and D’Amario [15] assessed the left atrial pressure with an implantable biosensor (V-LAP) in patients with heart failure, and confirmed that left-sided filling pressure is related to pre-symptomatic hemodynamic changes and congestion, with up to 21 days in advance of the clinical congestion symptoms (dyspnea, weight and heart rate change, peripheral oedema, and jugular venous pressure raise) which present late in the course of decompensation. NT-proBNP is a dynamic parameter, and its’ values are at the highest at admission, whereas at discharge, the values may decrease due to management.

These are the reasons we consider that NT-proBNP at admission, “specifically secreted from the cardiac chambers in response to volume and pressure overload leading to increased wall tension” [12], reflects the congestion of up to 3 weeks before [14,15] at its highest value, and may be a useful tool to predict 12-month mortality, whereas a re-evaluated and decreased (more or less) NT-proBNP at discharge (after proper management) is valuable in predicting rehospitalisations [12].

To the best of our knowledge, there is a single other prospective study, published in 2019, that had strictly selected patients with de novo HFpEF [25]. As a common denominator with the results of this study, we have also demonstrated the prognostic impact of eGFR (CKD-EPI), as it is one of the predictors for NT-proBNP above the threshold, but the above-mentioned study did not take into account NT-proBNP as a predictor of mortality. However, although the sex ratio (68% vs. 65.8% female), the age (73 ± 11 vs. 71.6 ± 9.1 years), and the incidence of arterial hypertension (all patients) were similar in both studies, the majority of the baseline characteristics of the patients are very different, as in our study, diabetes mellitus was more frequent (56% vs. 25.6%), the central tendency of SBP (185.4 mm Hg—mean vs. 140 mm Hg—median) and DBP at admission (99.2 mm Hg—mean vs 80 mm Hg—median) was higher, eGFR was less affected (66.5 mL/min/1.73 m^2^ vs. 50.2 mL/min/1.73 m^2^), and Hb was lower (12 g/dL vs. 13.5 g/dL), as the exclusion criteria were not as strict as ours. The study only explored the prediction capability of two different formulas of CKD-EPI, concluding that the creatinine-cystatin C CKD-EPI was associated with the mortality and rehospitalisation rate.

Among the enrolled patients in our study, 100% and 43.9% of them had arterial hypertension and CKD, respectively. Although eGFR is an independent predictor of plasma NT-proBNP levels, it seems that in patients with acute HF, the increased levels are mainly determined by acute cardiac stretch and secretion [26].

The wide range of the confidence interval (95% CI) of mortality risk at 12 months in our study (1.6–169) may be due to the low number of deaths in the low-set NT-proBNP group (<2910 pg/mL), and to the overall non-Gaussian distribution, with several outliers of the admission NT-proBNP (186 pg/mL to >30,000 pg/mL). It is possible that patients tolerate very high values of NT-proBNP, as an expression of the slow advance of the hemodynamic overload, with a concealed progression of HFpEF, before addressing to the physician.

NT-proBNP has been identified as a powerful independent mortality predictor in patients with chronic HF [27,28]. Higher values of NT-proBNP increase the risk of mortality for both new-onset HFpEF and HFrEF [29]. Salah et al. considered that a single measurement of NT-proBNP at admission or at discharge offers the same prognostic information for both HFrEF and HFpEF [10]. In another study, based on a cohort of 1088 patients with HFrEF or HFmrEF, it has been suggested that risk stratification can be made by splitting the patients into four groups, depending on the NT-proBNP levels (with the higher risk group for levels >1000 ng/L) [30]. Of note, the mortality at one year in our group of patients (15.4%) was comparable with the percentage found in a Spanish study published in 2019, which included 3288 patients with de novo HF irrespective of EF (13.2%), but did not focus the impact of NT-proBNP on mortality, as we did in our study [8]. In a subgroup of 744 patients with de novo HFpEF analysed in a recent Korean study, the mortality at one year was 17.2%, without presenting a threshold of NT-proBNP above which the risk of mortality increases [6].

Nevertheless, a limited number of studies have proposed a threshold for NT-proBNP that correlates with increased mortality. The populations included in these studies vary from patients with COVID (threshold of 88.64 pg/mL for in-hospital mortality) [31], COPD (threshold of 551.35 ng/L for in-hospital and 1-year mortality) [32], pneumonia [33] (threshold of 1,434.5 pg/mL for mortality at 30 days), severe obesity and stable HF (threshold of 879 ng/L for 5-year all-cause death) [34], to unselected subjects (150 pg/mL for mortality at ten years) [35]. Of relevance for our work, a Chinese study published in 2020 found that mortality was significantly higher in AHF patients with NT-proBNP > 2137 pg/mL and GFR < 61.7 mL/min/1.73 m^2^. Moreover, the difference in mortality was statistically significant between patients with eGFR > 61.7 mL/min/1.73 m^2^ and NT-proBNP above and under the threshold (the mortality rates at 18 months were 20% and 9.9%, respectively). Though the study focuses on the cumulative prognostic power of NT-proBNP and eGFR, the authors acknowledge that NT-proBNP has an independent value in predicting mortality, with an AUC of 0.65, slightly less than the value obtained in our study [36]. Although some of this increased mortality risk can be attributed to multiple other parameters, the measurement of BNP is easy and may provide a more useful prognostic tool.

Additionally, the threshold we identified is important because with a single measurement of NT-proBNP at admission for de novo and acute HFpEF, values less than 2910 pg/mL predict an extremely high (99.3%) likelihood of survival in the next 12 months.

**Study limitations.**

The study’s relatively small number of patients is the most important limitation. We acknowledge the fact that our findings may need external validation.

## 5. Conclusions

In patients admitted to the hospital for the first time with acute HFpEF, an admission NT-proBNP ≥ 2910 pg/mL predicted an adjusted 16-fold increased risk of all-cause mortality at 12 months, but not the number of rehospitalisations, whereas values less than 2910 pg/mL forecast an extremely high likelihood of survival (99.3%) in the next 12 months. It should be considered a useful prognostic tool in these patients, in addition to its utility in diagnosing heart failure.

## Figures and Tables

**Table 1:** Key baseline characteristics of the patients with HFpEF (*n* = 91).

Characteristics	Value
Number of subjects	91
Length of in-hospital stay, days, median (IQR)	7.5 (5)
Age at diagnosis, yo, mean ± SD (95% CI)	73 ± 10.6 (70.8–75.3)
Female gender, *n* (%)	62 (68.1%)
***Cardiovascular risk factors***	
Hypertension, *n* (%)	91 (100%)
Diabetes mellitus, *n* (%)	51 (56%)
Tobacco smoking (current or former), *n* (%)	25 (27.5%)
Hypercholesterolemia, *n* (%)	75 (82.4%)
BMI (kg/m^2^), mean ± SD (95% CI)	32.1 ± 6.3 (30.8–33.5)
***Previous medical history***	
CAD, *n* (%)	21 (23.1%)
MI, *n* (%)	13 (14.3%)
Stroke, *n* (%)	18 (19.8%)
Atrial fibrillation, *n* (%)	63 (69.2%)
Lung disease, *n* (%)	44 (48.4%)
Sleep apnoea, *n* (%)	12 (13.2%)
***Assessment at admission***	
Non-Invasive ventilation, *n* (%)	20 (22%)
Mechanical ventilation, *n* (%)	6 (6.6%)
Peripheral oedema, *n* (%)	53 (58.2%)
SpO_2_ (%), median (IQR)	89 (6)
HR (beats/min), median (IQR)	96 (55)
SBP (mm Hg), mean ± SD (95% CI)	185.4 ± 34.8 (178.2–192.7)
DBP (mm Hg), mean ± SD (95% CI)	99.2 ± 18.1 (95.5–103)
Serum sodium (mmol/L), median (IQR)	140 (5)
eGFR (mL/min/1.73 m^2^), mean ± SD (95% CI)	66.5 ± 28.8 (60.5–72.5)
Hb (g/dL), mean ± SD (95% CI)	12 ± 2.0 (11.6–12.4)
NT-proBNP (ng/L), median (IQR)	3074 (5241)
***Medication at discharge***	
ACEI, *n* (%)	46 (50.5%)
ARB/ARNI, *n* (%)	36 (39.6%)
Amlodipine, *n* (%)	51 (56%)
Antiarhythmics, *n* (%)	6 (6.6%)
Anticoagulants, *n* (%)	59 (64.8%)
Beta-blockers, *n* (%)	77 (84.6%)
Digoxin, *n* (%)	19 (20.9%)
Loop diuretics, *n* (%)	79 (86.8%)
MRA, *n* (%)	42 (46.2%)
Nitrates, *n* (%)	11 (12.1%)
Statines, *n* (%)	66 (72.5%)

MI: body mass index, CAD: coronary artery disease, eGFR: estimated glomerular filtration rate, Hb: haemoglobin, HR: heart rate, IQR: interquartile range, MI: myocardial infarction, SpO_2_: peripheral oxygen saturation, SBP: systolic blood pressure, DBP: diastolic blood pressure, NT-proBNP: N-terminal pro-B-type natriuretic peptide.

**Table 2:** Key baseline characteristics in the two NT-proBNP groups.

Characteristics	NT-proBNP < 2910 ng/mL	NT-proBNP ≥ 2910 ng/mL	*p*-Value
***Number (%)***	42 (46.2%)	49 (53.8%)	
Age (yr) mean ± SD (95% CI)	70.4 ± 10.2 (67.2–73.6)	75.3 ± 10.5 (72.3–78.3)	0.026
Male gender, *n* (%)	17 (40.5%)	12 (24.5%)	0.10
Smoking status, *n* (%)	15 (35.7%)	10 (20.4%)	0.10
***Medical history***			
Diabetes mellitus, *n* (%)	25 (59.5%)	26 (53.1%)	0.53
Hypercholesterolemia, *n* (%)	40 (95.2%)	35 (71.4%)	0.003
Atrial Fibrillation, *n* (%)	23 (54.8%)	40 (81.6%)	0.006
Coronary artery disease, *n* (%)	13 (31%)	8 (16.3%)	0.099
Lung disease, *n* (%)	22 (52.4%)	22 (44.9%)	0.47
Sleep apnea, *n* (%)	5 (11.9%)	7 (14.3%)	0.73
Number of comorbidities, median (IQR)	6 (2)	6 (3)	0.46
***At admission***			
Pulmonary edema, *n* (%)	15 (36.6%)	15 (31.3%)	0.59
Peripheral edema, *n* (%)	22 (52.4%)	31 (63.3%)	0.29
BMI (kg/m^2^), mean ± SD (95% CI)	32.7 ± 5.12 (31.1–34.3)	31.6 ± 7.1 (29.6–33.7)	0.43
SpO2 (%), median (IQR)	89 (7)	89 (7)	0.91
Heart rate (beats/min), median (IQR)	93.5 (45)	96 (58)	0.45
SBP (mm Hg), mean ± SD (95% CI)	189.5 ± 35.8 (178.4–200.7)	181.9 ± 33.9 (172.2–191.7)	0.3
DBP (mm Hg), mean ± SD (95% CI)	102.1 ± 19 (96.2–108.1)	96.7 ± 17 (91.8–101.6)	0.17
eGFR (mL/min/1.73 m^2^),mean ± SD (95% CI)	76.4 ± 29.9 (67.1–85.8)	58 ± 25.1 (50.8–65.2)	0.002
Haemoglobin (g/dL), mean ± SD (95% CI)	12.4 ± 1.5 (11.9–12.9)	11.6 ± 2.3 (10.5–12.3)	0.064
NT-proBNP (ng/L), median (IQR)	1529.5 (1698)	6700 (5298)	0.001
***Echocardiography at admission***			
Pulmonary edema, *n* (%)	15 (36.6%)	15 (31.3%)	0.59
Peripheral edema, *n* (%)	22 (52.4%)	31 (63.3%)	0.29
LVEF (%), mean ± SD (95% CI)	55 (7)	55 (10)	0.51
LVEDD (mm), mean ± SD (95% CI)	48.5 ± 5.2 (46.9–50.1)	46.9 ± 5.7 (45.3–48.6)	0.17
LV mass (g/m^2^), median (IQR)	125 (36)	122 (34.5)	0.63
LVOT VTI (cm), median (IQR)	18.85 (6)	18 (7)	0.5
TAPSE (mm), mean ± SD (95% CI)	20.9 ± 3.8 (19.7–22.1)	19 ± 3.7 (17.9–20)	0.017
TAPSE < 17 mm, *n* (%)	6 (14.3%)	15 (30.6%)	0.065
Systolic PAP (mm Hg),mean ± SD (95% CI)	41.1 ± 15.7 (36.2–46)	40.5 ± 14.4 (36.4–44.6)	0.83
Systolic PAP > 35, *n* (%)	26 (61.9%)	33 (67.3%)	0.58
IVC diameter > 21 mm, *n* (%)	18 (42.9%)	22 (44.9%)	0.84
IVC collapse < 50%, *n* (%)	11 (26.2%)	24 (49%)	0.026
LAVi (mL/m^2^), mean ± SD (95% CI)	47 ± 10.6 (43.7–50.3)	55.7 ± 12 (52.2–59.1)	0.001
LAVi > 34 mL/m^2^, *n* (%)	39 (92.9%)	48 (98%)	0.23
E/e’ratio	14.9 ± 4.9 (13.3–16.4)	14.2 ± 4.4 (12.9–15.5)	0.506
Stroke volume, median (IQR)	65.5 (26)	60 (33)	0.053
Right Atrium area > 18 cm^2^	24 (57.1)	39 (79.6)	0.021
In-hospital stay (days), median (IQR)	6 (4)	9 (6)	0.08
Rehospitalisation at 12 months, *n* (%)	18 (42.9%)	26 (53.1%)	0.33
Median time to readmission (days)	75 (105)	30 (45)	0.008
All-cause mortality at 12 months, *n* (%)	1 (2.4%)	13 (26.5%)	0.001
Time to death (days), median (IQR)	360	90 (252)	0.002
***Medication at discharge***			
ACEI, *n* (%)	19 (45.2%)	27 (55.1%)	0.253
Amlodipine, *n* (%)	26 (61.9%)	25 (51%)	0.409
ARB/ARNI, *n* (%)	21 (50%)	15 (30.6%)	0.084
Anticoagulants, *n* (%)	25 (59.5%)	34 (69.4%)	0.204
Antiarhythmics, *n* (%)	4 (9.5%)	2 (4%)	0.325
Beta-blockers, *n* (%)	34 (80.9%)	43 (87.8%)	0.149
Digoxin, *n* (%)	8 (19%)	11 (22.4%)	0.619
Loop diuretics, *n* (%)	34 (80.9%)	45 (91.8%)	0.028
MRA, *n* (%)	23 (54.8%)	24 (49%)	0.729
Nitrates, *n* (%)	4 (9.5%)	7 (14.3%)	0.445
Statines, *n* (%)	36 (85.7%)	30 (61.2%)	0.019

LVEDD, left ventricular end-diastolic diameter; TAPSE, tricuspid annular plane systolic excursion; PAP, pulmonary artery pressure; RA, right atrium; LVOT VTI, left ventricle outflow tract time velocity integral; HFR, heart failure readmission; SPAP, systolic pulmonary arterial pressure; IVC, inferior vena cava; LAVi, left atrial volume index.

## Data Availability

All data is on hospital records; all data is available on request.

## Supplementary Materials

The following supporting information can be downloaded at: https://www.mdpi.com/article/10.3390/jpm12060890/s1, Figure S1. NT-proBNP as a predictor of all-cause mortality at 12 months from the first episode of HFpEF (red), and its’ internal validation (blue) (Hanley and McNeil test, *p* = 0.82); Table S1. Transthoracic echocardiography at admission in 91 patients with HFpEF; Table S2. Univariate analysis for NT-proBNP ≥ 2910 pg/mL to-be-predictors; Table S3. The odds ratio for the increased NT-proBNP ≥ 2910 pg/mL.
