# Supplementary material for: B-Type Natriuretic Peptide at Admission Is a Predictor of All-Cause Mortality at One Year after the First Acute Episode of New-Onset Heart Failure with Preserved Ejection Fraction
Source: J Pers Med. 2022 May 28;12(6):890. doi: 10.3390/jpm12060890 (PMC9225135; doi:10.3390/jpm12060890)
Supplement: Supplementary file 1 [file jpm-12-00890-s001.zip › jpm-1728394-supplementary.pdf]

Journal of Personalized Medicine

Table S1. Transthoracic echocardiography at admission in 91 patients with HFpEF.

Characteristics	Value
Left ventricle	
LV end-diastolic diameter, mm, mean±SD (95%CI)	47.64±5.48 (46.50-48.78)
LVEF, vol %,median (IQR)	55 (10)
Septal s velocity, cm/s, mean±SD (95%CI)	6.22±1.20 (5.97-6.47)
Lateral s velocity, cm/s, mean±SD (95%CI)	7.35±1.30 (7.08-7.62)
Left ventricle mass, g/m², median (IQR)	122 (37)
LVOT VTI, cm, median (IQR)	18.60 (7)
Stroke volume, mL, median (IQR)	62 (26)
LA index volume, mL, mean±SD (95%CI)	51.67±12.11 (49.15-54.19)
E/e'ratio, mean±SD (95%CI)	14.51±4.61 (13.55-15.47)
E/e'ratio, median (IQR)	14 (6)
E/e'ratio>9, *n* (%)	82 (90.10%)
E/e'ratio>14, *n* (%)	47 (51.60%)
Septal e' velocity<7 cm/s, *n* (%)	65 (71.40%)
Lateral e' velocity<10 cm/s, *n* (%)	67 (73.60%)
Right ventricle, IVC, and right atrium	
Free wall RV S<9.5, *n* (%)	12 (13.20%)
TAPSE<17 mm, *n* (%)	23 (25.30%)
RA area over 18 cm², *n* (%)	63 (69.2%)
IVC over 21mm, *n* (%)	40 (44%)
IVC collapse<50%, *n* (%)	35 (38.50%)
PAPS, mm Hg, mean±SD (95%CI)	40.79±14.93 (37.68-43.90)
PAPS>35 mm Hg, *n* (%)	59 (64.80%)

HF: heart failure, Hb: Hemoglobin, HR: heart rate, IQR: interquartile range, IVC: inferior vena cava, MI: Myocardial Infarction, PAPS: systolic pulmonary artery pressure, SaO2: arterial oxygen saturation, SBP: systolic blood pressure, TAPSE: tricuspid annular plane systolic excursion, LA: left atrium, LV: left ventricle; LVEF: Left ventricle ejection fraction, LVOT VTI: left ventricular outflow tract velocity time integral; RA: right atrium, RV: right ventricle.

Table S2. Univariate analysis for NT-proBNP ≥ 2910 pg/ml to-be-predictors.

Crt	Variable	Omnibus test	Nagelkerke R Square	Hosmer and Lemeshow Test	*p*-value regression coefficient	AUROC	*p*-value AUROC
1	Age	0.024	0.073	0.309	0.030	0.647	0.016
2	Medical history of atrial fibrillation	0.005	0.109	-	0.034	0.634	0.028
3	eGFR (mL/min/1.73 m²)	0.002	0.137	0.768	0.004	0.669	0.006
4	TAPSE	0.016	0.083	0.194	0.021	0.658	0.009
5	LAVi (mL/m²)	0.001	0.173	0.920	0.001	0.716	0.001
6	IVC collapse<50%	0.025	0.072	-	0.028	0.614	0.06
7	Right Atrium area > 18cm²	0.020	0.077	-	0.023	0.612	0.06

**8**	Length of in-hospital stay	0.064	0.050	0.110	0.089	0.605	0.086
**9**	Stroke volume	0.087	0.042	0.040	0.094	0.618	0.054

Table S3. The odds ratio for the increased NT-proBNP ≥ 2910 pg/ml.

No.	Predictor	Odds ratio	Lower limit 95% CI	Upper limit 95% CI	*p*-value
1	Age > 73 years	3.712	1.557	8.851	0.003
2	Medical history of atrial fibrillation	3.671	1.428	9.441	0.007
3	eGRF ≤ 80 ml/min/1.73 m²	4.957	1.815	13.537	0.002
4	TAPSE ≤ 22 mm	5.984	1.969	18.183	0.001
5	LAVi > 54 ml/m²	5.208	1.943	13.961	0.002

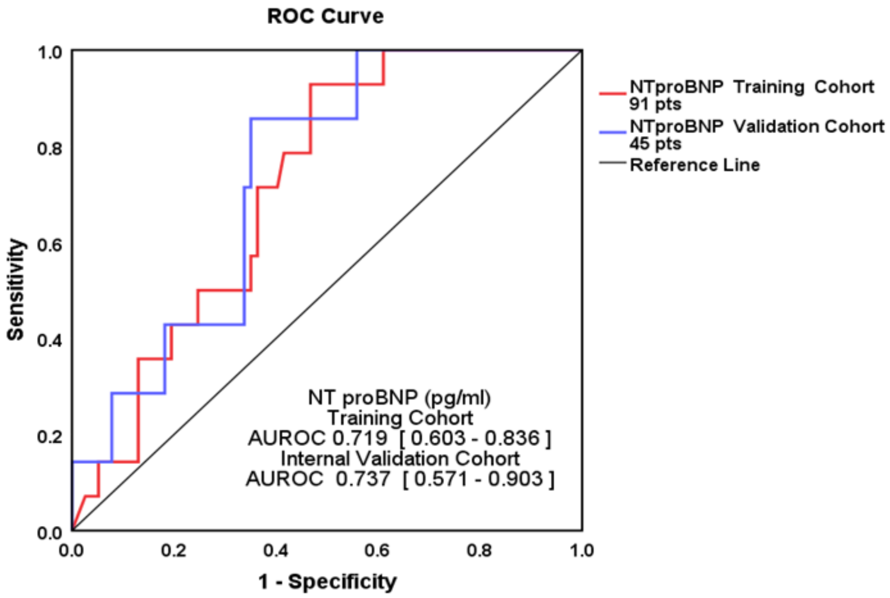

Figure S1. NT-proBNP as a predictor of all-cause mortality at 12 months from the first episode of HFpEF (red), and its' internal validation (blue) (Hanley & McNeil test, $p = 0.82$).
